# Identification of Two Early Folding Stage Prion Non-Local Contacts Suggested to Serve as Key Steps in Directing the Final Fold to Be Either Native or Pathogenic

**DOI:** 10.3390/ijms22168619

**Published:** 2021-08-10

**Authors:** Fernando Bergasa-Caceres, Herschel A. Rabitz

**Affiliations:** Department of Chemistry, Princeton University, Princeton, NJ 08544, USA; hrabitz@princeton.edu

**Keywords:** prion, folding, pathway, intermediate, molten globule, neuropathology

## Abstract

The initial steps of the folding pathway of the C-terminal domain of the murine prion protein *m*PrP(90–231) are predicted based on the sequential collapse model (SCM). A non-local dominant contact is found to form between the connecting region between helix 1 and β-sheet 1 and the C-terminal region of helix 3. This non-local contact nucleates the most populated molten globule-like intermediate along the folding pathway. A less stable early non-local contact between segments 120–124 and 179–183, located in the middle of helix 2, promotes the formation of a less populated molten globule-like intermediate. The formation of the dominant non-local contact constitutes an example of the postulated Nature’s Shortcut to the prion protein collapse into the native structure. The possible role of the less populated molten globule-like intermediate is explored as the potential initiation point for the folding for three pathogenic mutants (T182A, I214V, and Q211P in mouse prion numbering) of the prion protein.

## 1. Introduction

A direct link between the misfolding of the prion protein and several severe neurodegenerative conditions such as Creutzfeldt–Jakob and Kuru disease in humans, mad cow disease in cattle, and scrapie in sheep has been firmly established [1,2]. Specifically, the onset and development of the diseases was observed to be directly related to the accumulation in the brain of aggregates of a misfolded isoform of the native prion protein PrP^c^, called PrP^Sc^ [1,2]. The pathogenic isoform PrP^Sc^ has the same amino acid sequence as the native fold prion, but it bears considerably more ß-sheet secondary structure [3] and aggregates into oligomers and fibrils resistant to denaturation [4,5]. Thus, the elucidation of the mechanism by which the prion protein misfolds into its pathogenic isoform has become a prime issue. The importance of understanding the mechanisms underlying disease-related protein misfolding, aggregation, and fibrillization has become even more pressing as other neurodegenerative diseases such as Alzheimer’s [6] and Parkinson’s disease as well as Lewy dementia [7,8] have also been shown to display similar molecular mechanisms. Recently, it has also been suggested that the prion-like aggregation of the misfolded protein P53 might contribute to the onset and spreading of certain cancers [9,10].

This paper will consider the folding pathway of the prion protein into its native structure, while also giving a particular emphasis to issues associated with the pathogenic transition of the T182A, I214V, and Q211P mutants. A significant body of experimental evidence has shown that the prion pathogenic transformation is favored by mildly denaturing conditions, suggesting the involvement of molten globule-like states [11,12,13,14]. Direct experimental evidence indicates that: (a) the amyloid transition of the prion protein might be related to the presence of molten globule-like states that are prone to aggregation under pathogenic conditions [15,16,17,18,19], and (b) the branching point for the pathogenic transition is likely to be an on-pathway intermediate state [19]. Support for this hypothesis is also available from other protein systems such as β-microglobulin that display structural correlations between the molten globule state and the misfolded state [20,21]. However, the structure of such molten globule-like intermediate states of the prion protein remains unclear despite considerable efforts to elucidate them [22,23,24]. Thus, a topic of prime importance is the development of theoretical models to investigate the possible existence and structure of any intermediates along the folding pathway of the prion protein and consideration of their involvement in the pathogenic transition.

In this paper, we will apply the sequential collapse model (SCM) [25,26] to predict the earliest structure-forming events along the folding pathway of the murine prion protein *m*PrP(90–231). In the SCM, the multi-state folding process of proteins with a length of ~100–150 amino acids is initiated by the formation of a specific non-local contact between two distinct locations along the protein sequence called the primary contact [25,26]. The establishment of the primary contact that nucleates the folding process is hypothesized to considerably simplify the stochastic search leading to the native structure, by constraining the configurational options into smaller sets of potential dynamical trajectories. The primary contact would then constitute a set of emergent natural physical constraints that sidestep Levinthal’s paradox [26,27]. The SCM has been applied to investigate some general properties of the folding dynamics of pathogenic proteins in several contexts [28,29,30,31,32]. The main features of the SCM folding pathway for proteins ~100–150 amino acids long are explained in the Methods section.

The results presented in this paper include: (a) The prediction of two possible and competitive primary contacts nucleating the earliest stages of the folding pathway of the murine prion protein *m*PrP(90–231). The early populations of the non-local contacts are then explained to (b) produce a molten globule ensemble for *m*PrP(90–231) composed mostly of two different species, corresponding to the superposition of the molten globule-like intermediate states (MGLIS). The results are then applied to (c) explore the possible role in the pathogenesis of a change in the relative populations of MGLIS for three pathogenic mutations that are known to exist on the predicted primary contacts (T182A, I214V, Q211P) [33]. Additionally, (d) it is explained that the results suggest that the early non-local contact formation dynamics underlie the route to the prion pathogenic transition. Finally, (e) the model is shown to analytically provide for a quantitative estimate of the entropic barrier to the folding of the native prion protein, which is consistent with experimental observations.

Finally, it is important to point out that the investigation presented here deals only with the possible presence of a branching point towards the pathogenic structure along the folding pathway of the prion. It is not a study of possible interconversion mechanisms of the folded native structure as contemplated in many theoretical and experimental studies [34]. The possible existence of such an early branching mechanism from the molten globule does not necessarily imply that the same pathogenic structure could not be attained from the native fold. As the pathogenic isoform is a stable folded state of the prion, it is possible that it might be attained through more than one mechanism.

## 2. Results

### 2.1. The Murine Prion Protein mPrP

**Figure 1:**
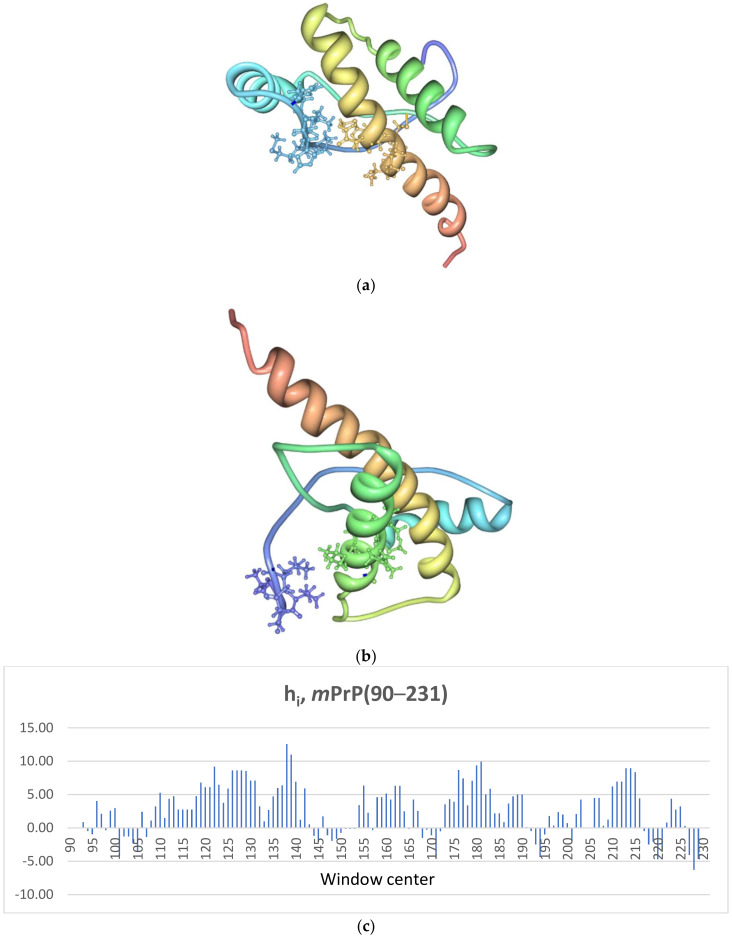
The location on the native structure [36,44] of *m*PrP(121–231) of the two possible primary contacts: (**a**) contact (138–213), and (**b**) contact (122–181) [45]; (**c**) the hydrophobicity coefficients h_i_ calculated employing a rolling 5-amino acid window (see Section 4.2 in Methods).

The murine prion protein PrP^C^ is a glycoprotein of 209 amino acids, (numbered 23–231 for human PrP) attached to the cell membrane by a glycosylphosphatidyl inositol (GPI) anchor at ser231 [35,36]. Its three-dimensional structure, shown in Figure 1, includes an autonomously folding C-terminal domain involving residues 121–231, *m*PrP(121–231) [12]. The structure of the C-terminal domain of PrP^C^ bears three α-helices (helix 1: D143-N153, helix 2: Q171-T192, and helix 3: E199-S226) and a short two-stranded antiparallel β-sheet [35]. 

The folding pathway of the C-terminal domain *m*PrP(121–231) has been explored employing a mutant F175W in order to have a fluorescence probe [37]. The folding reaction is very fast, with a half-time of τ ~170 μs at 4 °C. The reaction proceeds in almost two-state fashion, being one of the fastest naturally occurring protein folding reactions known to date. Experimental evidence on the human prion protein *hu*PrP(90–231) shows that there are intermediates along the folding pathway of prion proteins [16,38,39,40,41]. However, the experimental evidence is limited, and the structure of the intermediates is unclear. There is evidence for a very early intermediate in *m*PrP(121–231) [19]. This intermediate appears to be only marginally stable and involves few native-like contacts, with most of the chain folding in a two-state collapse-like mode [19,37]. 

The prion protein is very flexible, with regions of the protein chain apparently disordered in the native state [35,36]. Consistent with this flexibility, its sequence contains a larger percentage of relatively small amino acids including glycine (i.e., compared to most globular proteins) and less hydrophobic side chains as compared to compact globular proteins. In this sense, the prion sequence represents an intermediate stage between those of compact globular proteins and the vastly more flexible intrinsically disordered proteins (IDP) [42,43]. 

### 2.2. Primary Contacts for mPrP(90–231)

Following the methodology employed in the SCM before [25,26], our calculations searched for the most stable possible hydrophobic contacts between pairs of 5-amino acid segments i and j, located at a distance n_ij_ along the sequence such that 57 ≲ n_ij_ ≲ 100 amino acids. For this purpose, the hydrophobicity h_i_ of each segment was estimated and is shown in Figure 1 (see Section 4.3 in Methods for a complete explanation along with citations giving further details). Our results predict that there are two possible primary contacts (i.e., the earliest contacts along the folding pathway) as initiation points for the folding of *m*PrP(90–231) as shown in Table 1:

As shown in the table: (1) The best primary contact, PC1, in *m*PrP(90–231) is established between the two segments comprising residues ^136^PMIHF^140^, centered at I138, and ^211^QMCVT^215^, centered at C213. The 136–140 segment is located in the connecting region between strand 1 of the β-sheet and helix 1, and the 211–215 segment is included in the C-terminal region of helix 3. Contact PC1 has a stability of ΔG_cont_ ≈ −4.2 *k*T. (2) The second-best possible contact, PC2, appears between segment ^120^VVGLG^124^, centered at G122, a segment not included in the major secondary structure elements, and ^179^VNITI^183^, centered at I181. Segment 179–183 is included in the middle section of helix 2 in the native structure. Contact PC2 has a stability of ΔG_cont_ ≈ −2.1 *k*T. All other possible contacts have stabilities < *k*T and will not be considered in this analysis, as their populations are one order of magnitude, or more, smaller than those of the two most stable contacts. Based on the calculated stabilities, the populations of both contacts in the earliest stage of the folding pathway of *m*PrP(90–231) are then ~89% for PC1 and ~11% for PC2. 

The segments included in the two predicted primary contacts are highlighted on the native structure of mutant N173T (PDB: 1Y15 in mouse prion numbering) [36] in Figure 1. The two segments defining the dominant contact PC1 are close in the tertiary structure. The primary contact is relatively unstructured, enabling it to undergo further interactions in later folding phases. Thus, the segments involved in the primary contact are not necessarily precisely in coincidence in the native structure. However, they are generally close, with side chains within the Van der Waals interaction range, as they nucleate further structure attainment. The second-best contact (122–181) is not fully represented in the available NMR structure for *m*PrP(121–231) (PDB: 1AG2) [35], as it only includes residues V121-G124. It can be readily seen, however, on the structure of the mutant N173T [36] that the second-best contact, PC2, is not as close as the dominant one in the native structure, but still native-like.

### 2.3. Molten Globule State for mPrP(90–231)

**Figure 2:**
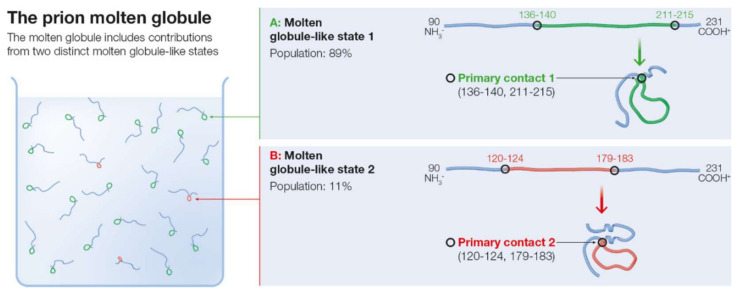
The molten globule of *m*PrP(90–231) includes contributions from two distinct MGLISs arising from the two primary contacts: (**A**) the MGLIS arising from the dominant primary contact, including helix 3; (**B**) the MGLIS arising from the second-best primary contact including helix 2 and, possibly, also helix 3.

Within the SCM model, the formation of a primary contact nucleates a molten globule-like intermediate state [25,26], as explained in the Methods section of the paper. The molten globule ensemble is then defined by the superposition of the MGLIS associated with each possible primary contact. Based on the predictions made above for the possible primary contacts, the molten globule ensemble of *m*PrP(90–231) is expected to include contributions from the MGLISs associated with each of the two predicted primary contacts. The expected composition of the molten globule ensemble is depicted in Figure 2. 

Because of the difference in population of the two possible primary contacts, the molten globule ensemble is heavily dominated by the native-like contribution of the MGLIS1 associated with PC1. Upon being nucleated by the primary contact PC1, the MGLIS1 could include the primary contact and several additional structural elements located within the segments 121–136 and 218–231 outside of this primary contact. Thus, the potentially folded region of MGLIS1 could include the C-terminal region of helix 3 in the native structure. While the formation of a well-defined molten globule, with considerable native-like structure is observed in other proteins, it is not evident experimentally for the prion protein [18].

The less populated PC2 nucleates a different MGLIS2, which, besides the primary contact, could include structure in the region 90–119. Region 90–119 is generally expected to be disordered in the native state. Structure could also form in the region 184–231, which includes helix 3 and helix 2 from its middle up to the C-terminal section in the folded structure. Because the population of MGLIS1 is predicted to be higher than that of MGLIS2, the population of native-like helix 3 should also be higher relative to that of helix 2.

The predicted molten globule ensemble correlates well with the limited experimental information that is available about the structure of the folding intermediates of *m*PrP(90–231). A molten globule state of *m*PrP(90–231) obtained through acid denaturation was observed to retain structure in the helix 2-helix3 region, while lacking any in the strand 1-helix 1-strand 2 segment [46]. The folding kinetics of human prion protein *hu*PrP(91–231), with a sequence very closely related to that of the murine protein, have been probed by temperature jump [47], and the folding nucleus was observed to contain elements of helices 2 and 3, which are both involved in the primary contacts predicted here. Additionally, hydrogen exchange of human PrP shows that a few residues clustered around region 179–214, which includes segments of both predicted primary contacts, have exchange rates sufficiently low to imply protection in the unfolded state [48]. Hydrogen exchange data of an on-pathway intermediate of the W145H/Y218W mutant of *m*PrP(90–231) are available [19]. For this mutant, the predicted native primary contact changes slightly from the one predicted here for the native sequence; the N-terminal segment remains the same, but the C-terminal segment becomes C214 to W218, centered at T216. This minor shift does not substantially affect the consideration of the molten globule above. The experiment yielded an intermediate with severe NMR line broadening in the helix 2-helix 3 region and with native-like elements, including amino acids at both ends of helix 3. The line broadening in the helix 2-helix 3 was expected to reflect the presence of the fluctuating folded-unfolded structure on the millisecond time scale. There was little protection elsewhere, including the N-terminal region where the P137-F141 segment is located, but protection of this segment is also low in the native state, so no definitive conclusions regarding its involvement in forming an early contact can be reliably reached. Consistent with the predictions made here, NMR chemical shifts in strand 1 of the β-sheet show significant displacement from negative values in the native state to values close to zero in the intermediate [19]. Helices 2 and 3 are also found to be critical in molecular dynamics studies of the prion interconversion due to the observed propensity of residues in helices 2 and 3 to interact to form β-sheet structures [49].

### 2.4. Is the Less Populated MGLIS the Branching Point for the Pathogenic Transition from the Molten Globule?

Several researchers have focused on the instabilities of helices 2 and 3 in the native state [50,51,52,53] and the significant number of pathogenic mutations that they can bear to propose that the transition from the native state is likely to be initiated from helices 2 and 3. Helices 2 and 3 remain partially folded in denatured states of the prion, while helix 1 is largely absent from such states [19]. This observation, combined with the additional stability of helix 1 as compared to helices 2 and 3 [54], has led some researchers to propose that the pathogenic transition from the native structure of the prion involves the destabilization of helix 1 [54], and conversely, that the stability of helix 1 constitutes a barrier to the pathogenic transition from the native state [55].

The presence in our predictions of a MGLIS defined by a significantly less populated primary contact in the earliest folding stages of the prion protein makes it important to investigate the possibility that the less populated MGLIS might constitute a pathway branching point leading to the pathogenic transition to PrP^Sc^. Consistent with this hypothesis, experiments carried out with deletion mutants of the human prion protein, showed that the region encompassing helix 2 is involved in the nucleation of amyloid fibrils [56]. Helix 2 peptides have also been shown to seed the formation of fibrils of full length *m*PrP with the same kinetics as full length *m*PrP^Sc^ [57], a property not shared by helix 3 seed peptides. In order to investigate this possibility, we will focus here on the three pathogenic mutations of *m*PrP that directly involve the segments defining the primary contacts that nucleate the MGLISs leading to the molten globule: T182A, I214V, and Q211P [33,58,59,60,61,62,63]. 

An atomic-level detailed analysis of the effects of these mutations on the stability of the two MGLIS states of the prion protein predicted here would require sophisticated molecular dynamics, which is beyond the scope of this paper. However, a similar approximation to the one made to calculate the stability of the primary contact can be made to estimate the effect of the mutations on the stability ΔΔG_MGLIS_ of the MGLISs. We will do so by employing empirically determined scales to estimate the combined effects of the mutation on the hydrophobic stabilization of the contact and the effect on the stability of helices 2 and 3 arising from the mutations. The approach is explained in the Methods section. The effect of the three mutations on the stability of the MGLISs is shown in Table 2.

An inspection of Table 2 shows that the effect of the three mutations is to increase the population of MGLIS2 with respect to that of MGLIS1. Mutation T182A does so by directly increasing the stability of MGLIS2, while mutations I214V and Q211P destabilize MGLIS1. The absolute value of the energy shift due to the change in secondary structure propensity is stronger than that calculated from hydrophobic stabilities alone for mutations T182A and Q211P. The T182A mutant suffers a considerable reduction both in secondary and tertiary native structure as compared to the native state [59,60], but not in the segment 182–196 corresponding to the primary contact [59]. Mutation I214V (I215V in human prion numbering) appeared in patients affected not only by Creutzfeldt–Jacob disease (CJD) but also in one case of Alzheimer’s disease, leading to the suggestion that it might be a marker for more general dementia conditions than just CJD [62]. Mutation Q211P (Q212P in human prion numbering) is remarkable because it induces significant adjustments in the prion structure, including partial disruption of helix 3, and an altered ability to bind copper, thus probably leading to alterations in copper homeostasis and redox activity in the affected cells [63]. 

The results presented here provide support for the hypothesis that mutations T182A, I214V, and Q211P become pathogenic by increasing the relative population of MGLIS2 with respect to MGLIS1. Contact PC2 for this mutation would become a gateway towards the pathogenic structure. In contrast, contact PC1 would constitute an example of what has been called in the context of the SCM “Nature’s Shortcut” to the native structure of the prion [26]. This result suggests that helix 2 might be critical to the interconversion process, as it appears partially folded in MGLIS2 but not in MGLIS1. This result is consistent with existing Φ-analysis of the aggregation process which suggests that α → β interconversion of helix 2 is an essential step in the formation of oligomers and fibrils [56]. Additionally, peptides mimicking helix 2 suffice to seed the short-time lag oligomerization of the native prion protein, while the same phenomenon was not observed when helix 3 mimicking peptides were employed [57].

### 2.5. Kinetics of the Two-State Collapse of the Prion Protein

In the SCM, the MGLIS collapses through two-state kinetics into the native topology [64]. The kinetics of the two-state collapse are described by the following equation:

ln κ_f_ ≈ ln g − RCO ΔG_conf_ (1)

where κ_f_ is the rate of two-state collapse, g is the characteristic diffusional frequency, RCO is the relative contact order (i.e., the average loop size of the final topology), and ΔG_conf_ is the entropic free energy cost of folding.

Equation (1) provides an analytical procedure to determine the size of the configurational barrier to the two-state collapse ΔG_conf_ from the observed two-state rate [28], such that

ΔG_conf_ ≈ (16.1 − ln κ_f_)/RCO (2)

The observed apparent two-state rate of collapse of *m*PrP(121–231) has a value of ln κ_f_ ≈ 8.65 [37]. Then, the application of Equation (1), employing the value of RCO = 0.104 obtained for *m*PrP(121–231) (PDB file 1AG2) [65], yields the configurational entropic free energy change of ΔG_conf_ ≈ 42.6 Kcal/mole, which compares very well with the experimental value of ΔG_conf_ = ΔG_int_ + ΔG_nat_ ≈ 42.5 Kcal/mole [66] for the full native *m*PrP. This result is in excellent agreement with two-state folding and experimental data, and it confirms that the C-terminal domain mostly participates in the folding process, with the N-terminal domain remaining significantly disordered.

**Figure 3:**
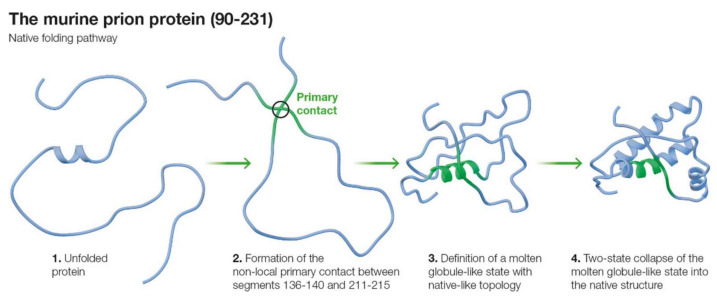
The native folding pathway of *m*PrP(90–231) predicted by the SCM. Helix 3 appears structured in MGLIS1 due to its participation in the primary contact. Stochastic search for the native topology is the rate limiting factor between the MGLIS (step 3) and the final native structure (step 4).

The complete native-like predicted folding pathway of the prion protein is shown in Figure 3.

**Figure 4:**
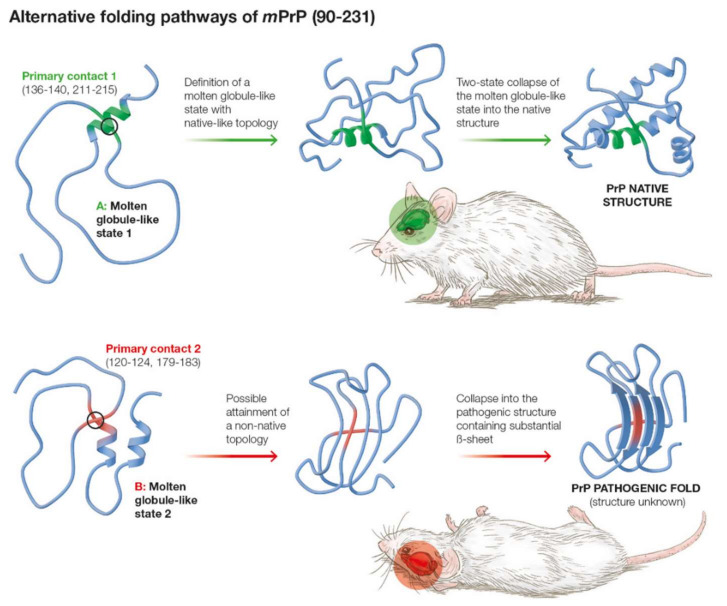
The alternative folding pathways with the less populated one possibly leading to pathogenesis. The schematic representation of the pathogenic topology and final fold is illustrative.

Several pathogenic prion mutations exist whose effect on the overall stability of the prion protein is only ~1–3 Kcal/mole [67]. This result is somewhat puzzling as, on the basis of simple transition-state theory, the kinetic separation between PrP^c^ and PrP^Sc^ is estimated to be ΔΔG_conf_ (PrP^c^ → PrP^Sc^) ~36–39 Kcal/mole in order for the relative populations to be consistent with the clinical evidence (1). Thus, based on simple transition-state theory we expect ΔG_conf_(PrP^Sc^) ~1.9·ΔG_conf_(PrP^c^), corresponding to a rate of folding difference such that κ_f_(PrP^Sc^)/κ_f_(PrP^c^) ~0.15. As explained above, within the SCM, the activation barrier to folding contains two distinct physical elements: (a) the configurational cost of burying a significant fraction of the amino acids into attractive interactions, and (b) the configurational activation barrier emerging from the complexity of the correct chain topology from the unfolded state, where higher complexity is understood as reflected in the higher fraction of long-range contacts in the native structure (i.e., higher contact order). Thus, it is possible within the SCM that ΔG_conf_(PrP^Sc^) ≈ ΔG_conf_(PrP^c^) and that the difference in folding rates between the pathogenic and the native forms is explained by the topological factor b) alone. Then, the contact order of PrP^Sc^ must be around RCO(PrP^Sc^) ≈ 1.9 RCO(PrP^c^) ≈ 0.2. This value compares well with naturally occurring proteins ranging between 0.1 and slightly larger than 0.2. Moreover, it is well established that the pathogenic PrP^Sc^ has a much larger β-sheet content (42%) than native PrP^c^ (3%) [68,69], and contact order values of β-sheet rich proteins tend to be higher than those of helical proteins [70]. Thus, a natural conclusion of the present analysis is that topology is the key kinetic protective factor that prevents most prion proteins from folding into the pathogenic PrP^Sc^ form. Finally, because the pathogenic transition in the current model requires the conformational search for the pathogenic topology described above, it is reasonable to expect that the proposed branching transition from MGLIS2 to the pathogenic structure takes place relatively late along the overall folding process as suggested by experimental data [19]. Figure 4 presents a schematic illustration of the pathogenic mechanism suggested by our results.

## 3. Discussion

The hypothesis that there is a pathogenic pathway to PrP^Sc^ through a molten globule-like state is broadly supported by a number of experimental observations. Thus, it is important to investigate the structure of such a molten globule state in order to understand the nature of the pathogenic transition. The current work predicts that two different molten globule-like states coexist within the molten globule for the murine prion protein *m*PrP(90–231). One of the two possible MGLISs is nucleated by a native-like contact and has the highest predicted population. The second possible MGLIS is also native-like but much less populated. The kinetics of formation of the dominant MGLIS and its progression towards the native structure were then investigated and shown to provide a strong consistency test for the model. Finally, it was also explained that the pathogenic mutations T182A, I214V, and Q211P stabilize the non-native MGLIS, suggesting a possible involvement of such a state in the pathogenic pathway associated with this mutation. The investigation of the pathogenic mutations required the consideration of the effects of secondary structure propensity in promoting the formation of primary contacts, which represents a significant predictive enrichment of the SCM model. 

The results presented here strongly suggest that the pathogenic effects of mutations T182A, Q211P, and I214V arise due to a shift in the relative population of native and non-native early non-local (i.e., primary) contacts. This hypothesis could be confirmed experimentally, for example, by altering the relative primary contact populations through directed mutation. It is important to bear in mind that the etiology of prion diseases is complex and multi-factorial [1,2,71]. This is clear from observation of the different progression of prion misfolding-related disease in several animal species [71]. Thus, while the results presented here aim to provide a significant additional perspective towards understanding the underlying molecular mechanisms at play, they should be viewed as a piece in a complex physiological puzzle. 

If proven correct, the results presented here could provide a new avenue for the development of therapeutic drugs. For example, a specific molecule could be sought that attaches to the segments that define PC2, thereby aiming to interdict in the formation of MGLIS2 and re-establish the native equilibrium between MGLIS1 and MGLIS2 within the molten globule. Such a folding interdiction of the target region (FITR) concept was proposed before within the SCM model for SARS-CoV-2 [72]. In particular, since the model predicts the specific segments that define PC2, it provides for natural templates to start the search for such an inhibitor of its formation, consisting of the peptides VVGLG or VNITI, corresponding to the protein segments that define PC2. There are many factors that go into the discovery of an effective therapeutic drug, and this natural suggestion or those possibly derived from it would also need to meet the host of criteria involved. The search for therapeutic drugs for prion diseases is an active field [60,73,74], including molecules aimed against the late stages of the folding process [75].

Furthermore, if our hypothesis is confirmed, it possibly could reveal a similarity of the pathogenic transition of the prion protein and that of the Parkinson’s disease-related protein α-synuclein. Although α-synuclein appears generally disordered both in vitro and in vivo, it is well established that there exist non-local contacts within the disordered state that are neuroprotective [76,77,78]. The relative populations of non-local contacts for α-synuclein were studied in previous work [32], and it was found that there is a dominant non-local contact and smaller populations of less stable ones. The results obtained here for mPrP(9.231) suggest that it could also be that a shift in the relative populations of the early non-local contacts of the prion protein similarly triggers agglomeration and pathogenesis. Recent experimental evidence indicates that the pathogenic transition of α-synuclein is dependent on the release of non-local interactions involving the N-terminal region [79]. The hypothesis that a common mechanism might underlie the pathogenic transition of the prion and α-synuclein could be investigated experimentally by altering the relative population of the non-local contacts of both proteins through directed mutation. If the hypothesis is confirmed, such equivalent mechanisms for the pathogenic transition of the prion protein and that of α-synuclein would point to the existence of a common intramolecular origin for at least some prion diseases and synucleinopathies. This, as yet, conjectured result would provide support for the commonality in the onset and development of several devastating neural diseases that has been suggested on the basis of broad biomedical considerations [80]. 

Further work will strive to investigate the effect of other pathogenic mutations in the folding pathway of the prion protein, thereby aiming to elucidate whether a common set of similar mechanisms underlies the pathogenic transition into PrP^Sc^. Finally, the identification of early non-local contacts that are critical to drive the folding of the prion protein into native or pathogenic structures could also provide insight into the search for potential inhibitors of misfolding by suggesting target regions along the sequence that are critical to reduce the population of misfolded proteins.

## 4. Methods

The physical basis of the SCM and its most up-to-date formulation have been recently explained in full detail [26,32]. Here, only a brief summary of the main concepts that are relevant to the issues investigated in the present paper is presented.

### 4.1. The Multi-State Folding Pathway of Small Proteins in the SCM

The SCM considers early non-local contacts based on the entropy of formation of the resultant protein loops and the hydrophobic stabilization energy of the protein segments that define the contacts. The SCM has successfully predicted many of the observed features of protein folding pathways at low resolution [26]. Within the SCM, the folding of proteins with a length of ~100–150 amino acids such as *m*PrP(90–231) is nucleated by the formation of specific early non-local contacts, called primary contacts, that define the earliest folding phase. Primary contacts form at an optimal distance that, following previous work [32], we calculated for mPrP(90–231) to be n ≥ n_op_ ≈ 57 along the sequence of amino acids determined from excluded volume considerations and polymer statistics [81]. The same kind of analysis showed that each primary contact in a long protein tends to define an autonomous folding region [82]. For a given protein sequence, there might be several possible stable primary contacts accordingly nucleating multiple parallel folding pathways [83]. Within the SCM, only those primary contacts that are native-like in the 3D structure lead to native-like folding pathways [26,82]. Because, at most, two simultaneous primary contacts can be established in proteins of a length of ~100–150 amino acids, most of the tertiary structure contacts will still be defined by contacts at a shorter range established in later folding phases. The nucleation by an early primary contact has been referred to within the model as “Nature’s shortcut to protein folding” [26].

It is important to bear in mind that the SCM is concerned with the optimal sizes of loops in the fluctuating unfolded chain rather than with the loops connecting secondary elements in the fully folded protein. Finally, because proteins longer than ~100 amino acids do not generally undergo complete two-state collapse 26but rather fold through multi-step pathways, consistent simple physical reasoning implies that there is an upper limit to the size of the primary loop that can successfully lead to the native SCM folding pathway of ~100 amino acids.

The concept of folding nucleated by non-local contacts is not exclusive of the SCM, having arisen earlier in the context of the diffusion-collision model [84], the loop hypothesis [85], and the energy landscape picture [86]. It also has appeared in simulations of the transition state of two-state folding proteins [87]. Additionally, protein topology has been considered an essential element of the folding mechanisms in a number of theoretical efforts [88,89,90,91,92]. The particular feature in the SCM is that the early non-local contacts are highly specific as in the loop hypothesis [85], and the SCM provides the means to determine their location from primary sequence information [25,26].

### 4.2. The Molten Globule

In the SCM, formation of a primary contact (i.e., a non-local early hydrophobic cluster) in a protein of ~100–150 amino acids nucleates the MGLIS. The MGLIS includes: (1) structured elements of the regions close to the segments defining the primary contact [25], located mostly within the N- and C-terminal ends of the protein, and (2) an open and fluctuating loop encompassing the regions of the sequence comprising the two segments defining the primary contact. The properties of the MGLIS listed below are close to those experimentally observed for the molten globule state [93]:

(a) The MGLIS has a primary loop in a fluctuating but generally open conformation due to the need to minimize ∆*G*_loop_ in the early folding stages.
(b) The MGLIS shows structural fluctuations on a larger scale compared to the native state because of the unfolded primary loop.
(c) The overall dimensions of the MGLIS are larger than those of the native protein because of the open primary loop.
(d) The extra volume of the MGLIS with respect to the native state is mostly water due to the open conformation of the loop.
(e) Side chains in the primary loop of the MGLIS retain much of their torsional freedom, because the loop is not a fully folded structure.

If several primary contacts arise, the set of possible MGLISs will appear experimentally superimposed in the molten globule ensemble. 

### 4.3. Determining the Primary Contact 

Based on the model presented in the previous sections, whether there is a non-local contact in an otherwise unfolded state is dependent upon the stability of the potential contact candidates at loop lengths of n ≥ n_op_ amino acids. In the SCM, the stability of a contact formed by the number n_cont_ of amino acids, ΔG_contact_(n_cont_, n_loop_), can be written as

ΔG_contact_(n_cont_, n_loop_) ≈ ΔG_int,H_(n_cont_) + ΔG_loop_(n_loop_) + ΔG_cont,S_(n_cont_) (3)

Here, ΔG_loop_ represents the entropic free energy cost of the loop as discussed in Section 4.1. The term ∆G_int,H_ denotes all the enthalpic interactions that help stabilize the contact, possibly including hydrophobic interactions, van der Waals interactions, hydrogen bonds, disulfide bonds, and salt bridges [94], and its value satisfies ∆Gint < 0. The term ΔG_cont,S_ > 0 represents the entropic cost of constraining the side chains of the amino acids defining the contact such that the contact is stable, and it opposes contact formation. A segment-specific determination of the value ΔG_cont,S_(n_cont_) for a given contact would require detailed MD techniques. However, a heuristic estimate can be made from earlier work within the SCM which showed that the average entropic cost of folding per amino acid for a sample of thirteen proteins was ΔG_folding/residue,S_ ≈ 0.85 *k*T/residue [28], and the maximum was ΔG_folding/residue,S_ ≈ 1.09 *k*T/residue. As these are estimates for the entropic cost for folding per residue of complete proteins that include highly buried as well as flexible exposed regions, it is then reasonable to expect that the entropic cost of a contact-forming region must be closer to the highest calculated values for ΔG_folding/residue,S_. Here, we will assume that ΔG_contact,S_(n_contact_) for a contact including n_cont_ amino acids is approximately ΔG_folding/residue,S_ determined by the number of residues defining the contact, such that ΔG_cont,S_(n_cont_) ≈ 1.09 n_cont_. This result is clearly an approximation but suffices for establishing a cut-off in the number of possible contacts that is consistent with the available experimental data.

Hydrophobic interactions are well understood to constitute the main driving force of the folding process [95]. Additionally, it is widely expected that secondary structure formation can contribute to the stability of early contacts [96]. Other interactions such as disulfide bonds and salt bridges are also understood to form later along the folding pathway [94]. Thus, for an early contact forming from the unfolded state, we can take ∆G_int_(n_op_) ≈ ∆G_hyd_(n_op_) + ∆G_helix_(n_op_), where ∆G_hyd_(n_op_) represents the stabilizing effect of hydrophobicity in the early contacts, and Equation (3) can be written as

ΔG_contact_(n_cont_, n_loop_) ≈ ΔG_hyd_(n_cont_) + ∆G_helix_(n_cont_) + ΔG_loop_(n_loop_) _+_ ΔG_contact,S_(n_contact_) (4)

There are no highly stable secondary elements in the intermediate states of the prion protein, so Equation (4) can be written as:

ΔG_contact_(n_cont_, n_loop_) ≈ ΔG_hyd_(n_cont_) + ΔG_loop_(n_loop_) + ΔG_cont,S_(n_cont_) (5)

Since the hydrophobic stabilization energy of the contact ΔG_hyd_ is determined by the hydrophobicity of the segments involved, the hydrophobicity values h_k_ are obtained from the Fauchere–Pliska scale [97] and assigned to each residue in accordance with previous calculations within the SCM. 

Because the amino acid side chains are significantly larger than the typical peptide bond length, early contacts between two hydrophobic amino acids will inherently involve segments including several amino acids adjacent to the initial contact. The stability of this early hydrophobic contact will determine where the folding process is initiated. This picture is not unlike the zapping model of Dill et al. [98]. Here, the typical early contact segment size will be taken to be ~5 amino acids in line with previous calculations within the SCM [25]. Also, a value of zero is assigned to the hydrophobicity of the end residues. Then, the hydrophobicity h_k_ of each residue is added over a segment contact window of five amino acids centered at residue i, resulting in a segment hydrophobicity h_i_,_5_ (a value of ~0.45 is equivalent to a change in energy of *k*T, and the margin of error is ~0.1 *k*T [96]). 

In order to determine the best contact, the h_i,5_ values of a segment centered at residue i are added to the h_j_ values of a segment centered at residue j, located at a distance n_ij_ at least n_op_ amino acids apart along the sequence, no longer than the maximum loop length of ~100 amino acids, to give a contact propensity of ΔG_cont_(n_cont_, n_loop_) ≈ *k*T {−(1/0.45)(h_i,5_ + h_j,5_) + [3/2 ln n_ij_] + 10.9}, where the term [3/2 ln n_ij_] is the classical Jacobson–Stockmeyer approximation to the entropic cost of loop closure [99]. We consider any two segments centered at less than 5 amino acids to represent the same contact-forming region, as there must be some flexibility as to the precise location of relatively loose hydrophobic contacts. 

### 4.4. Calculating the Effect of Mutations of the Primary Contact on the Stability of the MGLIS

The effect on the stability of the MGLIS, ΔG_MGLIS,_ of any point mutation M is difficult to calculate precisely in the absence of detailed structural information. In any case, precisely obtaining such an estimate would require sophisticated molecular dynamics [44] that are outside the scope of this paper. However, in order to estimate whether the effect on the stability of the MGLIS of a mutation in the primary contact, a simpler heuristic approach in line with the methodology explained above can be applied. Such a heuristic approach is consistent with the SCM’s main contention that the earliest folding steps can be determined through coarse-grained thermodynamic analysis and do not depend critically on the atomic detailed nature of the interactions involved [25]. We will consider two main effects of the mutation: a) the change in hydrophobicity, and b) the effect on formation of secondary structure, that is, helices 2 and 3. Then, the overall change in stability, ΔΔG_MGLIS_, upon mutation can be written as

ΔΔG_MGLIS_ ≈ ΔΔG_MGLIS,hyd_ + ΔΔG_MGLIS,__α_ (6)

where ΔΔG_MGLIS,hyd_ is the hydrophobic change in free energy upon mutation of the primary contact, and ΔΔG_MGLIS,__α_ is the change in free energy produced by the change in helical propensity of the primary contact upon mutation. 

With respect to the hydrophobic change in free energy, ΔΔGMGLIS,hyd, for mutations located on the primary contact, we closely follow the methodology described in Section 4.3. Then, the change in hydrophobic stability becomes ΔΔG_MGLIS,hyd_ ≈ ΔΔh_i,5_(M), that is, the result of calculating the difference between the hydrophobicity for the native primary contact and that for the same contact bearing mutation M.

An approximation to ΔΔG_MGLIS,__α_ will be made here in the same semi-empirical spirit as applied in estimating the hydrophobic stability of the primary contacts by employing the O’Neil–DeGrado scale [100] for the helix-forming tendencies α_i_ of the amino acids involved in close interactions. The scale is based on the stabilization energies of contacts between highly helical segments in dimers. This circumstance is an idealized physical situation unlikely to exactly resemble most primary contacts. At the level of resolution sought here, it is useful to provide a heuristic estimate of the mutation’s effect. Then, for a mutation M that replaces amino acid i for amino acid j, we can write ΔΔG_MGLIS,__α_ (M) ≈ αi_I_ − α_j_, where the αi_I_ coefficients are the O’Neil–DeGrado values for the free energy change upon replacement of glycine by amino acid i in a helical contact [100]. 

## Figures and Tables

**Table 1:** Predicted primary contacts for *m*PrP(90–231) (in *k*T).

Primary Contact	Stability	Native-like
PC1 (138 on 213)	−4.2	Yes
PC2 (122 on 181)	−2.1	Yes

**Table 2:** Estimated change in stability (in *k*T) of the corresponding MGLIS for mutations T182A, I214V, and Q211P, located in the two predicted primary contacts of the prion protein. Mutation T182A increases the stability of MGLIS2, while mutations I214V and Q211P reduce the stability of MGLIS1.

Mutant	MGLIS	ΔΔG_MGLIS,hyd_	ΔΔG_MGLIS,__α_	ΔΔG_MGLIS_
T182A	MGLIS2	−0.1	−1.1	−1.2
I214V	MGLIS1	0.6	0.2	0.8
Q211P	MGLIS1	−0.9	4.5	3.6

## Data Availability

Not applicable.

## Abbreviations

*m*PrP(90–231)	Murine prion protein (90–231)
SCM	Sequential collapse model
MGLIS	Molten globule-like state
